# Cortical complexity in eating disorders: a systematic review and qualitative synthesis

**DOI:** 10.1007/s00406-025-02001-3

**Published:** 2025-04-30

**Authors:** Enrico Collantoni, Gianni Pessotto, Valentina Meregalli, Christopher R. Madan, Alessandro Miola, Giammarco Cascino, Alessio Maria Monteleone, Angela Favaro

**Affiliations:** 1 Department of Neurosciences, University of Padua, Via Giustiniani, 2 – 35128 Padua, Italy; 2 Padua Neuroscience Center, University of Padua, Padua, Italy; 3 Department of General Psychology, University of Padua, Padua, Italy; 4 School of Psychology, University of Nottingham, Nottingham, UK; 5 Department of Medicine, Surgery and Dentistry, Scuola Medica Salernitana, University of Salerno, Salerno, Italy; 6 Department of Psychiatry, University of Campania L. Vanvitelli, Naples, Italy

**Keywords:** Eating disorders, Anorexia nervosa, Neuroimaging, Cortical complexity, Gyrification

## Abstract

**Background:**

Eating disorders (EDs) are complex psychiatric conditions with neurodevelopmental and neuroprogressive underpinnings. Altered cortical morphology, including gyrification patterns, may reflect these processes, offering insights into EDs pathophysiology.

**Objective:**

This systematic review and qualitative synthesis aimed to describe available neuroimaging findings on cortical complexity, including gyrification and sulcal morphology, in individuals with EDs compared to healthy controls (HC).

**Methods:**

Thirteen studies, including 525 patients with anorexia nervosa (AN), 69 patients with bulimia nervosa (BN) and 478 HC, were reviewed. Data on local gyrification index (LGI), sulcal morphology, and related cortical measures were systematically analyzed to identify consistent patterns of brain alterations.

**Results:**

A consistent reduction in LGI across frontal, temporal, and parietal regions was reported in patients with acute AN compared to HC. These findings suggest the presence of an altered cortical folding in AN, with alterations that may partially normalize following weight restoration. Studies on bulimia nervosa (BN) are limited, with findings remaining inconclusive. Emerging metrics, such as absolute mean curvature and fractal dimension, provide further insights but lack methodological consistency across studies.

**Conclusions:**

Altered cortical folding patterns, particularly decreased gyrification, may reflect neurodevelopmental disruption and the state-dependent effects of malnutrition in AN. Future research should focus on longitudinal studies and standardized neuroimaging methods to clarify these findings and expand knowledge on BN and binge eating disorder (BED).

**Supplementary Information:**

The online version contains supplementary material available at 10.1007/s00406-025-02001-3.

## Introduction

Eating Disorders (ED) are psychiatric disorders with a complex and multifactorial etiology, typically emerging during adolescence or young adulthood, and disproportionately affecting females [36]. Over the past decade, extensive research has aimed to elucidate the intricate neurobiological mechanisms underlying these conditions. However, findings remain inconclusive, and further efforts are needed to fully elucidate the pathophysiological correlates of brain alterations in affected individuals.

So far, research mainly focused on anorexia nervosa (AN), identifying a wide range of functional and structural brain changes associated with its acute phases [8]. One of the main challenges in evaluating the neurobiology of AN lies in the complex interplay of neurodevelopmental and neuroprogression processes [15]. AN emerges during adolescence, a critical period of neurodevelopment marked by substantial cortical remodelling, which may render individuals more vulnerable to both environmental and biological stressors. Malnutrition and the stress associated with the disorder are thought to disrupt these developmental processes further. Although evidence directly linking neurodevelopmental abnormalities to AN is limited, studies have pointed to potential early-life risk factors, such as adverse childhood experiences and genetic predispositions, that may interfere with typical brain development [6, 17]. Emerging literature, including large-scale neuroimaging studies, suggests that early disruptions in cortical morphology could heighten vulnerability to developing AN [12] [16]. However, this hypothesis requires further corroboration, particularly given the limited availability of longitudinal studies examining brain differences before the disorder's onset [12]. In the realm of neuroprogression, our understanding of AN remains limited, primarily due to the scarcity of long-term longitudinal studies. Preliminary findings indicate a potential association between the progression of the disorder toward long standing forms and distinct alterations in brain functionality and morphology [4]. Structural neuroimaging studies have demonstrated widespread cortical alterations in individuals with AN [21]. A pivotal investigation by the ENIGMA consortium revealed significant reductions in cortical thickness, subcortical volumes, and cortical surface area among a large cohort of patients with AN. These morphometric changes are closely linked to body mass index (BMI) and appear to improve with nutritional status, suggesting a state-dependent nature [35]. However, further research is needed to fully understand the dynamics and potential for recovery of these cortical alterations. Kaufmann et al. [20] conducted a longitudinal study assessing cortical and subcortical brain volumes at three distinct time points, revealing that the recovery of brain volumes after weight gain depends on the treatment phase, with more pronounced changes observed during the initial stages. Additionally, age was shown to significantly influence recovery trajectories, with younger patients exhibiting more substantial restoration. This evidence suggests that the capacity for structural cerebral restitution in AN may reflect the brain's inherent plasticity, being influenced by age and neurodevelopmental pathways. 

Although more limited, literature has also explored cortical morphology in other ED, such as bulimia nervosa (BN) and binge eating disorder (BED), suggesting that structural grey matter alterations may play a role in the neurobiology of these clinical conditions. In BN, research has indicated cortical thinning in areas like the frontoparietal cortex and the posterior cingulate cortex, which are associated with attention and control processes [3]. Moreover, in both disorders, research observed an increase in grey matter volume of the medial orbitofrontal cortex, an area involved in reward processing and the anticipation of pleasant stimuli [32]. These findings correspond with the dysregulated eating behaviours seen in individuals with BN and BED, where deficiencies in inhibitory control and altered reward processing may contribute to binge eating patterns.

Recently, advancements in neuroimaging have significantly expanded our capacity to explore cortical morphology through diverse computational methods and parameters, enabling nuanced interpretations of pathophysiological process impact at this level [8].

An important source of information, in addition to volume and cortical thickness measurements, may come from the study of cortical folding patterns. Numerous studies have demonstrated that the evaluation of cortical folding is crucial for gaining insights into brain development and organization, illustrating the interplay among genetic, neurodevelopmental, and environmental factors [1]. In eating disorders, alterations in cortical folding may stem from disruptions in early brain development or the effects of malnutrition associated with the disorder. Research focusing on sulcal morphology and gyrification supports these observations [10]. Such alterations, already implicated in conditions like schizophrenia and autism, may indicate impaired neural connectivity or cortical plasticity [31]. This knowledge enhances our understanding of the structural foundations of eating disorders and could aid in identifying biomarkers for disease progression or treatment efficacy.

Importantly, research on cortical gyrification patterns is currently limited partly due to the difficulty of capturing the complexity of cortical folding with a single index. Another significant challenge in studying cortical folding lies in interpreting its changes over developmental phases and how pathological processes may impact them [25].

The most commonly used index to study cortical folding patterns is the local gyrification index (LGI) [16], which is defined as the ratio between the inner folded contour and the length of the coronal outline. This index is thought to remain stable from the early postnatal period onward, providing a reliable representation of early neurodevelopmental processes. However, it's important to note that cortical morphology undergoes significant transformations during adolescence, characterised by a progressive flattening due to concurrent reductions in sulcal depth (SD) and expansions in sulcal width (SW). Some studies indicate that these changes in sulcal morphology during brain maturation likely underlie the age-related constancy of the gyrification index, with sulcal widening potentially compensating for the decrease in sulcal depth [10, 22].

Beyond LGI, other measures of cortical complexity have emerged as valuable tools for exploring the intricacies of cortical folding. One such measure is absolute mean curvature (AMC), which reflects the degree of bending of the cortical surface, allowing for the distinction between sharper and flatter cortical patterns [21]. Additionally, fractal dimension (FD) is an increasingly popular metric for assessing cortical complexity [5, 9]. This index provides a mathematical index that quantifies the self-similarity and intricacy of the brain's folded structure, making it a useful tool for capturing cortical changes. Studies have shown its sensitivity to structural brain alterations in many neurological and psychiatric conditions like Alzheimer's disease, schizophrenia, and multiple sclerosis, providing insights into both neurodevelopmental and degenerative processes [26]. Evaluating these mechanisms and parameters in ED, and in particular in AN, is crucial, given the profound morphological anomalies at the cortical level and the role of neurodevelopmental and neuroprogressive processes in its neurobiology.

Therefore, this review aims to assess the current state of research regarding the study of cortical convolutive patterns, their complexity, and sulcal morphology, providing insights into the structural brain alterations associated with these clinical conditions. Additionally, to better integrate and interpret the existing findings, we will conduct a qualitative synthesis of studies examining cortical gyrification in AN.

## Methods

### Literature search

The literature search for this study was conducted on January 24, 2024, using three distinct digital databases: PubMed (pubmed.ncbi.nlm.nih.gov), SCOPUS (www.scopus.com), and EMBASE (https://www.elsevier.com/products/embase). The search strategy employed the following key terms: ("eating disorders" OR "anorexia nervosa" OR "bulimia nervosa" OR "binge-eating disorder") AND ("brain gyrification" OR "cortical folding" OR "gyri*" OR "sulc*" OR "cortical complexity"). These terms were explored within the titles, abstracts, and keywords of all articles available in the databases, from their inception up to the search date. This approach, while comprehensive, may have introduced bias against studies not utilising these specific terms in their titles, abstracts, or keywords. This systematic review followed a pre-defined protocol registered in Open Science Framework (https://osf.io/pvr8k/) and adhered to the Preferred Reporting Items for Systematic Reviews and Meta-Analyses (PRISMA) statement [29].

### Study selection

**Fig. 1:**
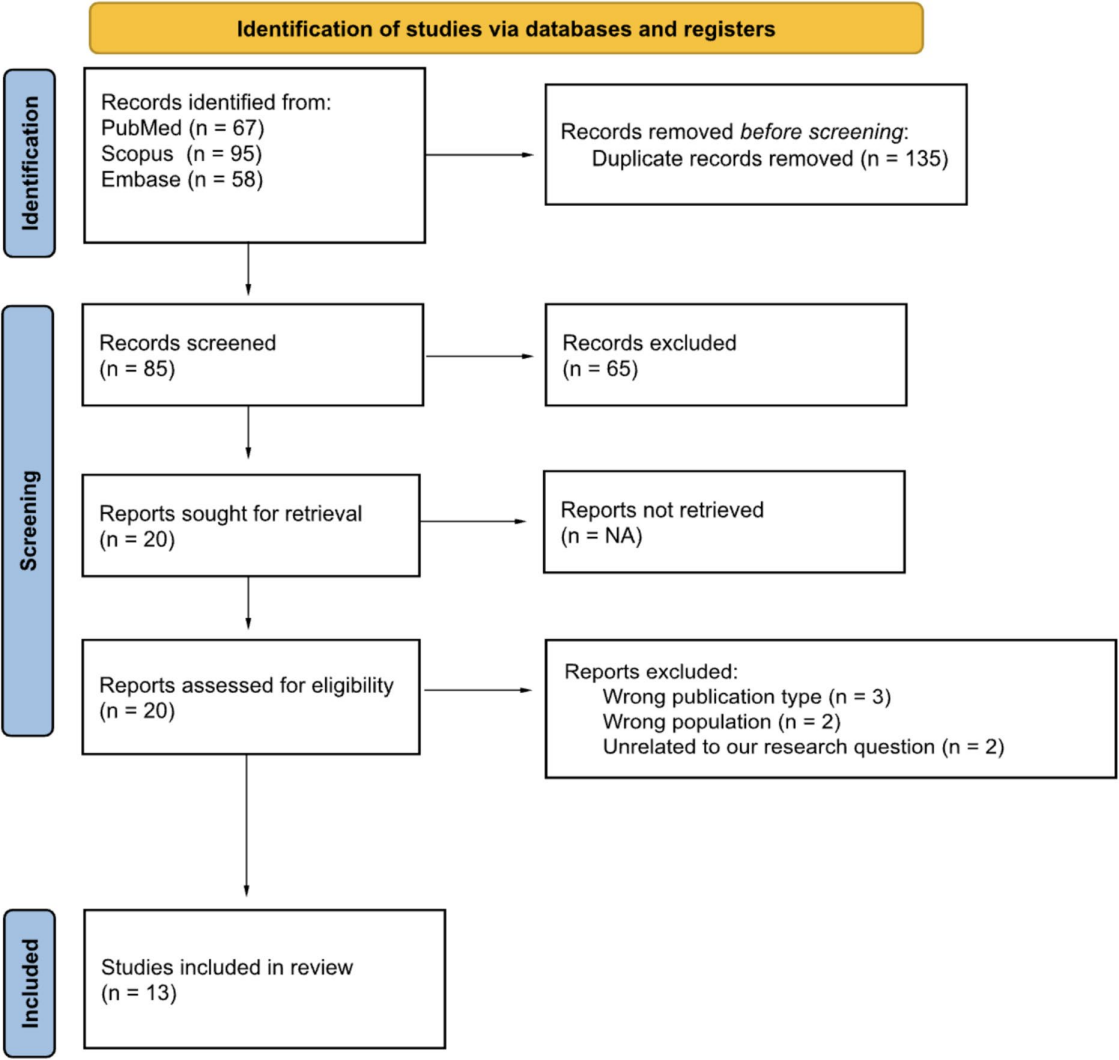
The Preferred Reporting items for Systematic Reviews and Meta-Analyses (PRISMA) flow-chart

After removing duplicates, three authors (VM, GP and AM) independently performed a preliminary screening of the search results by examining the titles and the abstract of the articles. Any discrepancies during this selection phase were addressed with input from a fourth author (EC). The inclusion criteria for the review were pre-defined before the search was conducted but were explicitly applied during the full-text evaluation stage to ensure their relevance and alignment with the study's objectives. The inclusion criteria were as follows: (i) studies must include a group of patients with a diagnosis of AN, BN, or BED (ii) must include a measure of cortical folding pattern or complexity (e.g. local gyrification index, fractal dimension, sulcal morphology, absolute mean curvature), (iii) must include a health control group, (iv) must be published in a peer-reviewed journal, and (v) must be written in English. During the screening process, a total of 65 studies were excluded for the following reasons: 23 studies did not include patients with anorexia nervosa, 6 were of an unsuitable publication type (e.g., reviews, single case studies, corrigenda), 1 was an animal study, 1 did not include a control group, and 34 extracted brain measures that were not relevant to the study’s objectives (e.g., fMRI, CT, or volumetric data). These exclusion reasons have been summarized in Fig. 1 to provide transparency in the study selection process.

### Data extraction and synthesis

Data were independently extracted into tables by four authors (AC, AM, GP, VM). The methodological quality of the studies was assessed by these authors, initially working separately and then collaboratively. From all included articles, we extracted the following information when possible: (i) characteristics and demographics of the participant samples (diagnosis, numerosity, age, sex, BMI), (ii) features of the disorder and treatment (age of onset, illness duration, treatment status, and setting), (iii) features of image acquisition and processing (image resolution, processing software), (iv) cortical measures and type of analysis (whole brain vs. ROI), and (v) main findings. This information is summarized in Tables 1 and 2.

**Table 1:** Demographics and characteristics of the included studies

Author Year	Study Design	Country	Diagnostic criteria	Groups	N (F%)	Age: Years (σ)	BMI (σ)	Medicated	Setting	Age onset: Years (σ)	Ill duration: Months (σ)
[16]	cross-sectional	Italy	DSM-IV	AN AN rec HC	38 (100%) 20 (100%) 38 (100%)	26.1 (7.2) 26.3 (7.1) 25.3 (6.3)	15.8 (1.8) 19.6 (1.6) 21.7 (2.9)	14/38 4/20 0/38	Out	18.3 (5.1) 17.7 (3.2)	78.6 (81.3) 45.7 (65.0)
[33]	cross-sectional	Germany	DSM-IV	AN HC	26 (88%) 36 (92%)	23.0 (5.0) 23.7 (2.1)	17.0 (1.5) 21.7 (1.5)	1/26 0/36	NA	NA	NA
[2]	cross-sectional; longitudinal	Germany	DSM-IV	AN (t1) AN (t2) AN rec HC	87 (100%) 57 (100%) 58 (100%) 142 (100%)	16.5 (3.1) 16.1 (2.0) 22.0 (3.3) 19.4 (4.3)	14.7 (1.4) 18.8 (1.2) 20.6 (1.7) 21.2 (2.3)	2/87 2/58 0/142	NA	15.2 (2.9)	15.0 (22.0)
[27]	cross-sectional	Canada	DSM-5	AN AN rec HC	23 (100%) 24 (100%) 24 (100%)	29.6 (8.2) 27.0 (6.1) 25.0 (5.7)	16.0 (1.4) 20.9 (1.8) 22.3 (2.2)	14/23 9/24 0/24	NA	15.0 (5.1) 15.0 (2.9)	158.4 (12.0) 115.2 (12.0)
[11]	cross-sectional;	Italy	DSM-5	AN (t1) ANrec (t1) HC AN (t2) norec AN (t2) rec	38 (100%) 20 (100%) 38 (100%) 24 13	26.1 (7.2) 26.3 (7.1) 25.3 (6.3) 25.5 (6.8) 26.7 (7.5)	15.8 (1.8) 19.6 (1.6) 21.7 (2.9) 14.9 (1.8) 16.2 (1.6)	14/38 4/20 0/38	Out	18.3 (5.1) 17.7 (3.2) 20.8 (6.5) 17.1 (3.8)	78.6 (81.3) 45.7 (65.0) 40.0 (46.2) 101.2 (89.8)
[23]	cross-sectional	England	DSM-5	AN HC	46 (100%) 54 (100%)	27.5 (9.2) 26.4 (4.5)	15.7 (1.4) 21.5 (2.0)	23/46 0/54	NA	NA	136.7 (110.6)
[28]	cross-sectional	Germany	DSM-5	AN AN rec HC	34 (100%) 24 (100%) 41 (100%)	23.8 (4.3) 27.1 (7.0) 23.6 (3.0)	16.1 (1.4) 20.6 (1.3) 22.3 (2.4)	0/34 0/24 0/41	Mixed	NA	74.4 (66.0) 62.4 (51.6)
[5]	cross-sectional	Italy	DSM-5	AN AN rec BN HC	22 (100%) 10 (100%) 24 (100%) 35 (100%)	28.6 (9.8) 25.5 (6.7) 27.2 (7.1) 26.8 (5.2)	16.4 (1.6) 19.8 (1.5) 22.3 (2.9) 21.1 (2.0)	0/22 0/10 0/24 0/35	Out	NA	12.5 (8.6) 5.33 (4.7) 8.31 (7.5)
[9]	cross-sectional	Italy	DSM-5	AN AN rec HC	38 (100%) 20 (100%) 38 (100%)	26.1 (7.2) 26.3 (7.0) 25.2 (6.7)	16.0 (1.8) 19.6 (1.6) 21.6 (3.0)	14/38 4/20 0/38	Out	18.3 (5.0) 17.7 (3.2)	78.6 (81.2) 45.7 (65.0)
[10]	cross-sectional	Italy	DSM-5	Site 1 AN AN rec HC Site 2 AN HC	38 (100%) 20 (100%) 38 (100%) 16 (100%) 16 (100%)	26.1 (7.2) 26.3 (7.1) 25.3 (6.3) 21.2 (4.3) 22.5 (2.4)	15.8 (1.8) 19.6 (1.6) 21.7 (2.9) 16.1 (1.2) 21.6 (2.4)	14/38 4/20 0/38	Out	18.3 (5.1) 17.7 (3.2) 20.2 (4.3)	78.6 (81.3) 45.7 (65.0) 8.8 (3.1)
[18]	cross-sectional	England	DSM-5	AN AN rec HC	57 (100%) 60 (100%) 70 (100%)	19.40 (2.8) 18.25 (3.5) 19.41 (3.3)	16.4 (1.4) 20.3 (2.4) 22.9 (3.4)	NA	NA	NA	44.3 (33.7) 44.9 (32.8)
[24]	cross-sectional	China	DSM-5	BN HC	45 (95%) 28 (93%)	24.67 (6.0) 25.93 (2.9)	21.4 (3.8) 20.9 (2.1)	0/45 0/28	Out	21.1 (5.1)	42.1 (48.3)
[7]	cross-sectional; longitudinal	Germany	DSM-IV	AN (t1) AN (t2) HC	38 (100%) 52 (100%)	15.3 (1.9) 15.5 (1.9) 15.7 (1.7)	− 2.7 (1.1)* 1.0 (0.5) 0.1 (0.9)	NA	In	14.1 (1.6)	12.6 (9.5) 16.2 (9.9)
Legend: AN: anorexia nervosa; BN: bulimia nervosa; HC: healthy control; AN rec: recovered AN; *BMI standard deviation score

**Table 2:** Main results of the studies included in the systematic review

Author, Year	Psychological and cognitive measures	Field Strenght	Software	Cortical measure	Analysis (whole brain vs. ROI)	Main findings
Favaro [16]	EHI, HSCL, EDI	1.5 T	FreeSurfer version 5.3.0	LGI	Vertex-wise comparison for LGI Regional comparison for FD	LGI: LGI: in Acute AN, overall significantly lower in both hemispheres compared to HC; also lower in specific areas including in the left hemisphere: precentral, postcentral, superior parietal, supramarginal, and rostral-middle-frontal gyri; in the right hemisphere: precentral and superior-frontal gyri. In recAN no significant differences compared to HCs. LGI clinical correlation: In acute AN, no significant correlation with BMI, weight loss, age of onset, or duration of illness in acute AN patients; A weak negative correlation between left hemisphere gyrification and duration of illness. Negative age correlations were found in both acute AN and HC, but no significant interaction between correlation and group was observed. Patients with poor outcomes showed significantly lower baseline gyrification compared to both HCs and recAN. LGI as outcome predictor: LGI were effective in predicting good outcomes in acute AN patients, with a high percentage of individuals correctly classified
Schultz [33]	BDI, EDI-2, EAT-26	1.5 T	FreeSurfer version 4.0.5	AMC	vertex-wise	AMC: significantly increased in a right premotor/dorsolateral prefrontal region in AN when compared to HC
Bernardoni [2]	EDI-2, BDI-II, SCL-90-R	3 T	FreeSurfer version 5.3.0	LGI AMC	Vertex-wise comparison	LGI: significantly reduced in patients with acute AN at T1 (acAN-T1) compared to HC. Individuals who underwent partial weight restoration (acAN-T2) exhibited a rapid normalization, reversing initial reductions. No difference between recAN and HCs. Normalization during weight restoration appears to be much faster than age-related changes in cortical folding AMC: widespread increased in acAN-T1. Following weight restoration (acAN-T2), AMC levels normalized, similar to LGI changes. No difference between recAN and HCs LGI and AMC Clinical correlations: Improvements in depressive symptoms or changes in leptin levels did not significantly influence the normalization of LGI or AMC during weight restoration. LGI and CT relationship: CT changes had a direct influence on LGI alterations in specific brain regions like the pre- and postcentral gyrus. Partial mediation analysis suggested that CT changes due to weight restoration partially drove the observed LGI increases
Miles [27]	EDE-Q 6.0	3 T	Freesurfer version 6.0.0	LGI	vertex-wise comparison	LGI: In recAN was significantly lower in cluster a L lateral orbitalfrontal cortex and a L superior frontal gyrus cluters compared to HC and significantly lower in cluster R medial orbitalfrontal cluster and a different L superior frontal gyrus cluster compared to acute AN In acute AN was significantly lower in cluster R postcental cluster compared to both HC and recAN
Collantoni [11]	HSCL, EDI, EHI	1.5 T	FreeSurfer version 5.3.0	LGI	vertex-wise comparison for LGI connectomics	Gyrification based networks: In acute AN compared to HC, no differences were detected in integration and segregation measures; SWI was significantly higher in Acute AN. In recAN compared to HC, No significantly differences Acute AN-t2 compared to recAN-t2: patients with poor outcome showed significantly higher clustering; at a regional level they exhibited higher normalized degree index in the inferior part of the right circular sulcus of the insula and higher normalized clustering of the left superior temporal sulcus. SWI was significantly higher in the good outcome group
Leppanen [23]	EDEQ, HADS, DASS	1.5 T	FreeSurfer version 6.0.0	LGI CC	Global and local cortical structures	LGI: significantly reduced in acute AN compared to HC in the L lateral frontal cortex, insula, superior temporal cortex and postcentral cortex; and in R lateral frontal cortex, insula and supramarginal gyrus CC: No significant differences found between acute AN and HC groups. LGI clinical correlation: Negative correlation between LGI in the postcentral cortex and duration of illness in acute AN patients
Nickel [28]	EDE, SCID I, SCID II, BDI-II, EDI‐2, STAI, MWT-B	3 T	CAT12 and MATLAB2013b	AMC, SD, FD	vertex‐ and region‐of‐interest‐wise level using statistical parametric mapping	AMC vertex wise: acute AN showed significantly lower AMC in left milddle temporal gyrus compared to HC ROI-wise: No significant differences SD: vertex wise: acute AN exhibited significantly lower SD as compared with HC in: right insula lobe, right calcerine sulcus, right superior temporal sulcus, and left rolandic operculum ROI-wise: acute AN revelead significantly lower SD in the right transverse temporal sulcus compared to HC and recAN FD: vertex wise: lower in acute AN than HC in: the left middle occipital gyrus and the right middle frontal gyrus. acute AN showed higher FD in the left precentral gyrus compared to HC ROI-wise: No significant differences
Cascino [5]	EDI-2	3 T	FreeSuerfer version 5.3.0	FD LGI	Vertex-wise comparison for LGI Regional comparison for FD	FD: no significant difference LGI: lower in bilateral superior frontal and caudal middle frontal gyri, left superior temporal and right precentral gyri in acAN compared to HC; no differences between recAN and HC; Compared to HC, BN showed lower LGI in the left medial OFC, but these differences were not significant after correction for multiple comparisons
Collantoni [9]	EHI, HSCL, EDI	1.5 T	FreeSurfer version 5.3.0	FD	regional comparison	FD: In acute AN, lower FD values were observed across various cortical areas compared to HC, particularly in regions spanning frontal, parietal, and temporal lobes bilaterally. No significant differences between patients of the restricting type and those of the binge-eating/purging type, or between patients taking antidepressants and those who were not. When compared to HC, recAN showed higher FD in the left superior temporal sulcus and the left subcentral gyrus and sulcus, and lower FD in the bilateral superior parietal lobule, left postcentral gyrus, right intraparietal sulcus, and bilateral parieto-occipital sulci. FD and clinical correlation: FD values were negatively correlated with age in all groups, with a stronger decline observed in acute AN patients, especially in frontal and parietal lobes. Positive correlations were found between whole-brain FD and BMI in acute AN patients, while negative correlations were observed between FD and illness duration in this group, and between FD and age of onset in recAN
Collantoni 2021	NA	1.5 T	FreeSurfer version 5.3.0	SW SD anterior–posterior gyrification gradient	Regional comparison for SW and SD regional comparison for gyrification gradient	Sulcal morphology: SW: Acute AN exhibited significantly greater sulcal width in both hemispheres in the Central, Post-Central, Parieto-Occipital, and Pars Marginalis of the Cingulate Sulci compared to HC; No significant differences were observed between recAN and HC. SD: Acute ANc showed increased depth in the L Pars Marginalis of the Cingulate Sulcus compared to HC; No significant differences between recAN and HC. Factor Analysis: Six factors were identified for SW and SD. Significant differences were found in specific factors between acute AN and both recAN and HW, indicating distinct patterns of sulcal morphology alterations in acute AN. anterior–posterior gyrification gradient: acute AN exhibited a similar pattern to HC, but with an overall reduced magnitude. Significant reductions were found in the Central Sulcus, Parieto-Temporal regions, and Frontal Lobe in acute AN compared to HC. Differences between shortAN (< 1 year) and longAN (> 1 year) SW: significantly higher in L and R Central and Post-Central sulci, in R Parieto-Occipital sulcus and in R Pars Marginalis of the Cingulate Sulcus in shortAN compared to HC; higher in R Post-Central sulcus and in Central, Parieto-Occipital and Pars Marginalis of Cingulate sulci bilaterally in longAN compared to HC. SD: higher in the left Pars Marginalis of the Cingulate sulcus in shortAN compared to HC; higher in the left and right Pars Marginalis of the Cingulate sulcus in longAN compared to HC Correlation with Clinical Indices: SD: Positive correlations were found between BMI and SD factors in shortAN. Negative correlations were found between disorder duration and specific SD factors in both short and long AN. SW: Positive correlations were found between disorder duration and SW factors in longAN. Negative correlations were found between disorder duration and specific SW factors in both short and long AN groups. Gyrification Gradient: negatively correlated with the duration of illness in the entire acute AN group and in longAN subgroup; negatively correlated with the age of onset in shortAN. An uncorrected correlation analysis revealed negative correlations between the first factor computed on SW and gyrification gradient in the whole acute AN sample and in longAN. Between the second factor computed on SW and gyrification in shortAN, positive correlations were found. Positive correlations were found between the second factor on SD and gyrification in the whole acute AN sample
Halls [18]	EDE-Q, NART, ADOS-2	3 T	Freesurfer version 6.0	LGI CC	vertex-wise comparison	LGI: Acute AN had reduced global LGI compared to recAN and HC, with significant reductions in the post-central and supramarginal gyrus. LGI clinical correlation: global LGI exhibited a negative correlation with age in acute AN CC: No group differences Correlation with ASD: No correlations between ASD traits and structural measures existed
Li [24]	DEBQ, EDI-I, EAT-26, BDI-II, SAS	3 T	Structural SPM12 functional fMRIprep and xcp-Engine	Mean Curvature, SD surface-based FC	vertex-wise for mean curvature and SD ROI to vertex and ROI to voxel for FC	Mean Curvaure: no significant between-group differences SD: significantly greater values in the right superior temporal gyrus and in the right medial orbitofrontal cortex in BN compared to HC
Collantoni [7]	EDI‐II, HAWIK‐IV or MWT‐B	3 T	Freesurfer version 7.1.1	LGI	vertex‐wise comparison connectomics	LGI based network: at baseline, acute AN had reduced global efficiency and SWI compared to HCs. The longitudinal comparison within the acute AN group showed no significant difference between baseline and follow-up. After partial weight gain, AN group exhibited no statistically significant differences in lower global efficiency and SWI compared to HC

### Study quality and results

The quality of the included studies was assessed with the Imaging Methodology Quality Assessment Checklist (adapted from [34]) (see Supplementary Material).

### Qualitative synthesis of Cortical Gyrification

A qualitative synthesis was performed on seven studies examining cortical gyrification differences between patients with acute AN and healthy controls. Thresholded cortical surface maps from each study were obtained and smoothed to create continuous surface representations. These surface renders were then overlaid to identify regions where gyrification differences were consistently reported across studies (e.g., [19]). The presented approach has several limitations, but is the current state-of-the-art. Here we considered three limitations in the reporting of published results: First, as the results of the several studies are quite distributed across the cortical surface [2, 7, 23], a table of peaks is not sensible for gyrification results. What would be ideal is an unthresholded cortical surface statistical map, which could then be combined with a sample size weighting with other studies. However, this quantitative statistical approach is not yet standardised or conventional in the structural MRI literature, as is now occurring in the fMRI literature [14, 30]. Second, even when affected regions are not overly distributed, the shape of the statistically significant region is often irregular, making the reporting of a peak less useful for meta-analytic purposes (e.g., [27]). Third, in studies of AN, it is common to include three groups of individuals: acute AN, recovering AN, and healthy control. As such, some researchers report aggregate statistics for differences in regional gyrification across the three groups, while some skip this step and focus on pairwise comparisons between groups. This variability in statistical reporting further complicates the possibility of comparing study results in a meta-analysis. Nonetheless, the figures that report the results can be visually combined, providing a less precise aggregation of results.

A similar qualitative synthesis could not be conducted for other cortical complexity parameters, such as fractal dimension, cortical curvature, or sulcal morphology, due to the limited number of studies and methodological heterogeneity. Variability in parcellation schemes and extraction methods precludes meaningful comparisons and highlights the need for standardized approaches in future research.

## Results

### Gyrification

#### Gyrification in acute anorexia nervosa

Seven of the retrieved studies analyzed gyrification in patients with acute AN, all consistently finding significantly decreased GI compared to HC. Specific reductions of LGI were observed in various cortical areas in patients with acute AN, particularly in regions spanning the bilateral frontal [5, 16, 23], left temporal [5, 23], bilateral parietal [23], right parietal [16, 18, 27], and bilateral insular [23] lobes. Using anterior–posterior gyrification gradient, Collantoni et al. [10] observed similar results, finding reductions in the central sulcus, parieto-temporal regions, and frontal lobe in patients with acute AN. Lastly, in a longitudinal study Bernardoni et al. [2] observed rapid LGI normalization after partial weight restoration in patients with acute AN. This normalization occurred more rapidly than age-related changes in cortical folding and resembled alterations seen in AMC.

#### Gyrification in Recovered anorexia nervosa

Regarding studies on patients who recovered from AN, few authors noted significant results [27]. described significantly lower LGI in patients who recovered from AN in a left frontal lobe cluster compared to HC and patients with acute AN,however, they noted lower values in a parietal cluster when comparing patients with acute AN to both patients who recovered and HC.

#### Qualitative synthesis of cortical gyrification in anorexia nervosa

**Fig. 2:**
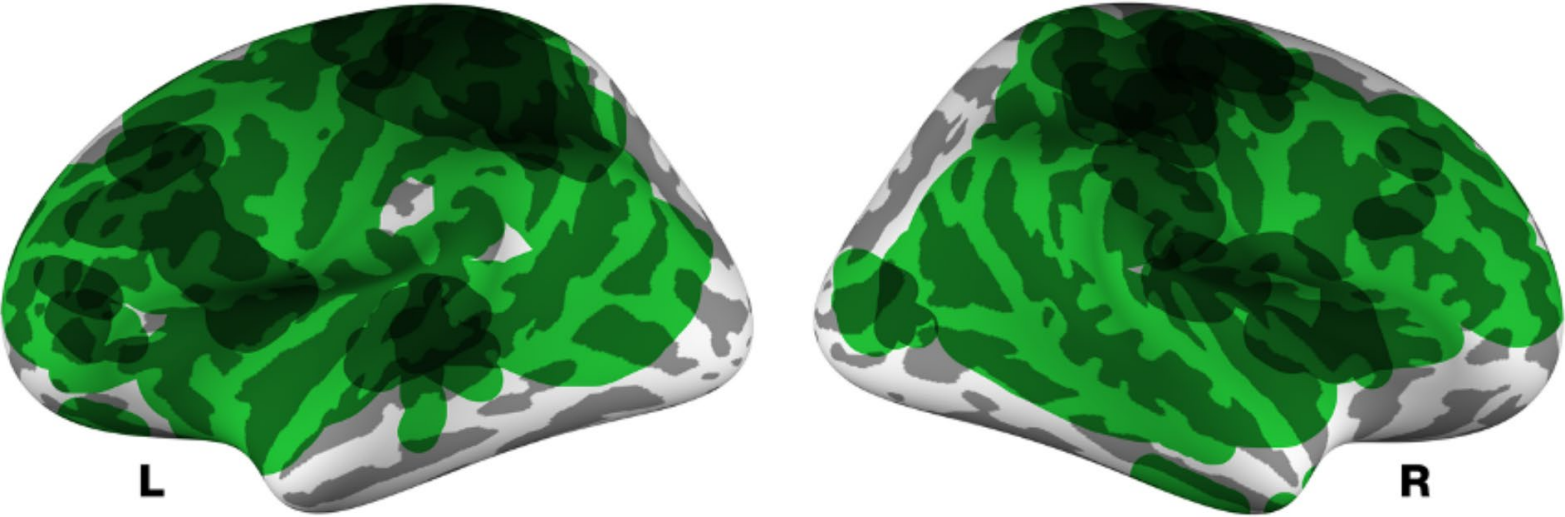
Qualitative synthesis of gyrification results reported in the seven studies reviewed. Light green regions were only reported as having gyrification differences due to anorexia in few studies, whereas dark regions were reported in several studies. See main text for further details

As shown in Fig. 2, gyrification of much of the lateral surface has been reported to be affected in AN, based on the results of Bernardoni et al. [2] and,Collantoni et al. [7]. The right superior parietal lobule was reported in most studies (including [7, 18, 23, 27]). Left superior parietal lobule effects of AN were reported in several studies as well [16, 23, 27]. Additionally, left prefrontal differences were also reported in multiple studies [5, 23, 27].

#### Gyrification in bulimia nervosa

Only one [5] of the retrieved studies examined gyrification in bulimia. Using LGI as measure the authors noticed reduced values of LGI in the left medial OFC in patients with BN compared to HC, but these differences were not significant after correction for multiple comparisons.

#### Associations with other morphological indices and clinical data

Two of the retrieved studies assessed correlations between gyrification and other morphological indices. Collantoni et al. [10] observed that the reduction in the gyrification gradient in patients with AN tends to localize in areas characterized by higher levels of sulcal widening, primarily confined to the central and post-central regions of the cortex. In their longitudinal evaluation, Bernardoni et al. [2] evidenced that cortical thickness alterations directly influenced LGI alterations in specific brain regions such as the pre- and postcentral gyrus. Furthermore, a partial mediation analysis suggested that cortical thickness changes due to weight restoration partially drove the observed LGI increases.

Five of the retrieved studies identified significant correlations between gyrification and clinical variables Collantoni et al. [10] found that the gyrification gradient negatively correlated with the duration of illness in all patients with acute AN and in those with a longer duration of the disorder, while it was negatively correlated with the age of onset in patients with a shorter duration of the disorder. Similarly, using LGI as a measure of gyrification, Leppanen et al. [23] also identified a negative correlation between LGI in the post-central cortex and illness duration in patients with acute AN,Additionally, Halls et al. [18] found that global LGI exhibited a negative correlation with age in patients with acute AN.

In a prognostic context, Favaro et al. [16] found that patients with poor outcomes exhibited significantly lower baseline gyrification compared to both healthy controls and patients who recovered from AN. Using a regression model, LGI proved effective in predicting favourable outcomes in patients with acute AN, achieving a high percentage of correct classifications.

#### Gyrification and graph theory

Two studies [7, 11] assessed graph measures in gyrification-based networks. The first study found higher Small World Index (SWI) values but no integration and segregation differences between acute AN patients and controls. In contrast, the latest study, which employed a longitudinal design, evidenced different results. In this study, the authors compared patients with acute AN at baseline and after a short-term weight restoration together and with a HC group. The results evidenced that patients with acute AN showed reduced SWI and global efficiency when compared to HCs, while after weight gain, patients with AN did not display statistically significant differences compared with their healthy counterparts. An association between the duration of the disorder and average clustering at baseline, and with local efficiency at follow-up was pointed out.

In both studies, networks were constructed using a correlation matrix of local gyrification indices across brain regions parcellated according to the Destrieux atlas. Binary adjacency matrices were generated, and network properties were analyzed across a range of connection densities, ensuring fully connected graphs for reliable comparison of measures such as the SWI. More specifically, both researches computed the SWI by comparing the characteristic path length and clustering coefficient of a graph with the corresponding values of null random graphs with the same number of nodes, edges, and degree distribution. A SWI > 1 indicated a network with relatively high segregation and integration compared to random null networks.

### Fractal dimension

Three studies analysed FD in patients with ED [5, 9, 28]. All included patients with AN and those who recovered from AN, while only Cascino et al. [5] also included patients with BN. Except for Cascino et al. [5], all the studies found significant results. Considering the comparison between AN and HC, Nickel et al. [28] observed lower FD values in acute AN in the left middle occipital gyrus and the right middle frontal gyrus. Conversely, higher values were reported in the left precentral gyrus. Similarly, Collantoni et al. [9] noted lower FD across various cortical areas in acute AN, particularly in regions spanning frontal, parietal, and temporal lobes bilaterally. Looking at the differences between patients who recovered from AN and HC, Collantoni et al. [9] found in recAN higher FD in the left superior temporal sulcus and the left subcentral gyrus and sulcus, but lower values in the bilateral superior parietal lobules, the left postcentral gyrus, the right intraparietal sulcus, and the bilateral parieto-occipital sulci. In addition, considering correlations with clinical data, Collantoni et al. [9] noticed that FD values were negatively associated with age in all groups, with a stronger decline observed in acute AN, especially in frontal and parietal lobes. In acute AN group there were positive correlations between whole-brain FD and BMI, while negative correlations were observed between FD and illness duration and between FD and age of onset.

### Cortical curvature

Six of the retrieved studies examined cortical curvature in ED.

The three studies that used Mean Curvature as a measure of evaluation did not find any significant results. The remaining three studies [2, 28, 33] utilized AMC to analyse cortical curvature in patients with AN. Except for Schultz et al. [33], these studies also considered patients who recovered from AN.

Nickel et al. [28] observed significantly lower AMC in the left middle temporal gyrus in patients with AN compared to HC. Conversely, Schultz et al. [33] found significantly increased values in a right premotor/dorsolateral prefrontal region in patients with AN when compared to HC. Similar results were found by Bernardoni et al. [2] who marked a widespread increase of AMC in patients with acute AN. Notably in the same article the authors highlighted that AMC levels normalize following weight restoration, similar to LGI changes. Neither of the last two articles did not find any significant difference between patients who had recovered from AN and HC.

### Sulcal morphology

Three of the retrieved studies examined sulcal morphology in ED using SD, but only Collantoni et al. [10] also employed SW. Two studies involved patients with AN and those who recovered from AN, while Li et al. [24] included only patients with BN.

Considering the comparison between AN and HC, Collantoni et al. [10] found increased SD in the left pars marginalis of the cingulate sulcus and significantly greater SW in both hemispheres in the central, post-central, parieto-occipital, and pars marginalis of the cingulate sulci in patients with AN. Interestingly, prospectively observing how sulcal morphology varied according to the different stages of AN, they noticed a similar pattern of alterations in SD and SW across the groups of patients with different disorder durations. In contrast, Nickel et al. [28] found that patients with AN exhibited significantly lower SD in several brain regions, including the right insula lobe, right calcarine sulcus, right superior temporal sulcus, and left rolandic operculum, when analyzed vertex-wise. Additionally, ROI-wise analysis showed significantly lower SD in the right transverse temporal sulcus in patients with acute AN.

Assessing the patients in remission from AN, only Nickel et al. [28] observed any significant result, finding that patients who recovered had, like HC, significantly higher SD in the right transverse temporal sulcus compared to patients with acute AN when using a ROI-wise approach.

Examining the differences between patients with BN and HC, Li et al. [24] identified significantly greater SD in the right superior temporal gyrus and in the right medial orbitofrontal cortex.

### Quality assessment

All the retrieved studies demonstrated a low risk of bias; in fact, each study received a score of 8.5 or higher (see supplementary material for details). The parameters that most often did not achieve full marks were related to 'subjects,' particularly regarding the complete exclusion of any psychiatric disorders and relevant medical conditions in the healthy control group, which was not always fully ensured.

## Discussion

This review provides a comprehensive analysis of the existing literature on cortical gyrification, complexity, and sulcal morphology in EDs, with a more specific focus on AN. The findings reveal some consistent patterns and identify areas that warrant further investigation. Most studies examined the local gyrification index and consistently reported a decrease of this parameter in patients with acute AN compared to HCs, particularly in the frontal, parietal, and temporal lobes. These results, as highlighted in the literature review, are also supported by our qualitative synthesis approach, which we employed due to the inability to conduct a quantitative meta-analysis. However, due to the cross-sectional nature of these studies, it is unclear whether these alterations may reflect early neurodevelopmental abnormalities or processes specifically related to the acute phases of the disorder. Supporting the hypothesis that gyrification alterations may be state-dependent, observations in patients who have recovered from AN generally do not show significant differences in LGI compared to HCs. This is further supported by a longitudinal study demonstrating a rapid restoration of LGI following partial weight recovery, with normalization occurring more quickly than typical age-related changes in cortical folding [2]. Notably, the same study reported that changes in cortical thickness appeared to partially mediate the observed increases in LGI during re-nutrition. These findings suggest that cortical thickness alterations may influence gyrification in specific regions, such as the pre- and postcentral gyrus. However, this observation should be interpreted cautiously and does not imply a universally established causal relationship. While studies on mean curvature did not yield significant results, those examining AMC presented heterogeneous findings, likely due to the use of different computational methods [2, 28, 33]. The only study highlighting a reduction in AMC in patients with AN utilized a vertex-wise approach. In contrast, the studies showing increased AMC values employed a surface-based method using the FreeSurfer's calculation system. The relationship between longitudinal changes in AMC and the LGI highlighted in Bernardoni et al. [2] study is particularly compelling, as it demonstrates that these two indices capture distinct yet complementary aspects of cortical morphology. During the acute phases of AN, a decrease in gyrification combined with an increase in AMC suggests a deflated and sharper cortex. This pattern reverses during weight restoration, indicating the presence of reinflating mechanisms.

Another parameter that can be associated with alterations in cortical gyrification is sulcal morphology. Two studies focused on sulcal depth (SD) during the acute phases of the AN using a vertex-wise and ROI-wise approach. A study conducted by Nickel and colleagues revealed a reduction of SD primarily in the insula and in the temporal lobe, while another more recent evaluation found an increase in this index in the left Pars Marginalis of the Cingulate Sulcus compared to HCs [10, 28]. The study conducted by Collantoni and colleagues extended this analysis to also assess sulcal width (SW), identifying increased widening in several major cortical sulci [10]. Furthermore, by examining an anterior–posterior gyrification gradient, this study found that reductions in cortical folding tend to occur in regions where sulci are wider, suggesting a potential relationship between alterations in gyrification and sulcal morphology. Overall, these findings suggest that modifications in the cortical gyrification index may be linked to alterations in the balance between sulcal depth and width, driven by underlying pathophysiological mechanisms that warrant further investigation. The functional relevance of these cortical alterations remains an important area of investigation. Similar patterns of altered cortical folding, such as decreased gyrification and increased sulcal width, have been reported in other neurodevelopmental and neuropsychiatric conditions, including schizophrenia and autism, where they are believed to reflect disrupted neural connectivity and reduced cortical plasticity [25]. In AN, these morphological changes may arise from a combination of malnutrition and disrupted neurodevelopmental processes. Specifically, malnutrition-induced reductions in brain volume are likely to contribute to sulcal widening, which may subsequently influence gyrification patterns, as highlighted in previous studies [2, 10]. Understanding how these cortical alterations relate to cognitive function and their potential impact on AN symptoms remains a critical direction for future research.

In recent times, there has been significant interest in employing fractal dimension (FD) to assess cortical morphology [26]. This measure, traditionally used to characterize the complexity of natural structures, is now being applied to study brain complexity across various scales, from the molecular to the whole brain level [13]. The convoluted geometry of the cortex, which results from the contribution of different components such as the outer surface, the gray matter-white matter interface, and the cortical ribbon, makes it an ideal candidate for analysis using FD. Studies assessing this parameter in AN are not entirely comparable due to different computational methods and parcellation schemes. However, given the observed alterations in this index and the sensitivity it showed to key clinical parameters, such as BMI and disease duration, further research is crucial to increase methodological standardization and improve comparability and reliability across studies.

Recent evidence points to LGI as a potential prognostic marker in AN. For instance, a study by Favaro and colleagues revealed that patients with a poorer treatment outcome had a lower baseline gyrification index compared to both a healthy control group and a group of patients who had recovered from the disorder [16]. Additionally, using a regression model, the authors demonstrated that the gyrification index could predict better clinical outcomes.

These findings were supported by a more recent study that used structural covariance analysis with graph theory tools on the same experimental sample. This study showed that the poor outcome group had higher segregation measures and lower small-worldness, suggesting that reduced global integration might be associated with a lower response to treatment [11].

This reduced global integration in gyrification covariance networks was also found in a recent longitudinal study on adolescent patients with AN before and after inpatient treatment. The study highlighted lower global efficiency and small-worldness at baseline. Upon weight restoration, there was a trend towards normalization of these indices, which were no longer significantly different from the HC group. The same study also observed an association between segregation parameters, such as the clustering index, and the duration of the disorder at baseline [7]. The discrepancies in findings related to SWI and network efficiency may stem from differences in the studied populations, particularly regarding age and disorder duration. Younger patients with shorter illness durations may exhibit distinct cortical network configurations compared to adults with longer disorder duration, reflecting divergent neurodevelopmental trajectories. Furthermore, variations in the clinical stage at recruitment, such as the severity of malnutrition or the level of clinical stabilization, could also contribute to these inconsistencies. These findings emphasize the importance of considering developmental and clinical variables when evaluating structural network properties in AN. Interestingly, results obtained on network properties on the longitudinal sample align with a previous longitudinal study examining gyrification patterns [2], supporting the idea that alterations in this index may be state-dependent and specifically related to malnutrition phases. However, it should be noted that both studies were conducted on adolescent inpatients over a very short period (approximately three months). Consequently, the effects on adult patients with longer illness durations remain unknown and should be evaluated in future studies that better consider the role of neuroprogressive factors in the disorder's pathophysiology.

The fact that this review predominantly focuses on AN reflects the relative abundance of research in this clinical population rather than a deliberate omission of BN or BED. Regarding BN, current studies are few and do not provide significant evidence of altered cortical folding patterns in this disorder. Similarly, no studies to date have investigated cortical folding in BED patients, leaving significant gaps in our understanding of how these disorders might impact cortical morphology. The absence of studies on cortical complexity and gyrification pattern in these two clinical conditions represents a critical limitation in the field. Unlike AN, where cortical alterations may be influenced by state-dependent factors such as malnutrition and refeeding processes, BN and BED offer an opportunity to investigate cortical morphology in the absence of such confounding variables. This distinction could allow for a clearer examination of the relationship between cortical structure and the specific psychopathological and neurobiological traits of these conditions, including impulsivity, emotional dysregulation, and altered reward sensitivity. These traits are central to the pathophysiology of BN and BED and may interact differently with cortical morphology compared to AN, thereby highlighting the need for dedicated research in these populations.

This review has some limitations that should be acknowledged. Firstly, the heterogeneity in data reporting across the included studies precludes the possibility of conducting a quantitative meta-analysis. Secondly, most of the studies are cross-sectional, with only two conducting longitudinal analyses on adolescent patients with AN. Furthermore, the short duration of follow-up in these studies limits the ability to assess long-term changes in cortical morphology. Third, many of the studies included in this review have relatively small sample sizes, which can impact the statistical power and reliability of the findings. Fourth, the clinical variability inherent to patients with AN, such as differences in illness duration, severity, and treatment history, is not always adequately addressed in the studies. Fifth, a limitation of our study lies in the use of a dedicated quality assessment tool, which, while tailored to neuroimaging studies in eating disorders, may not fully capture all potential sources of bias inherent to complex studies in such heterogeneous clinical populations. Future efforts could benefit from further refinement and standardization of tools for systematic reviews in this field to improve the robustness and applicability of quality evaluations. Finally, the absence of standardized protocols for data analysis across studies can result in variations that complicate the synthesis of findings and the drawing of robust conclusions. A particular consideration should be given to the qualitative synthesis of gyrification data included in this review, which highlighted some intrinsic limitations in the general literature on studies analyzing cortical complexity with surface-based methods. These challenges underscore the difficulty in producing a quantitative synthesis of data related to AN. Methodological hurdles include differences in the tools used to measure cortical complexity, the variability in sample sizes and imaging protocols, and the inconsistent reporting of results across studies.

In conclusion, this systematic review points out notable findings in cortical complexity and gyrification patterns in AN. Alterations in local GI, AMC, SD, and SW are consistently observed during the acute phases of the disorder, indicating state-dependent alterations related to malnutrition phases. Longitudinal studies suggest partial normalization of cortical gyrification with weight restoration in adolescent patients, emphasizing the potential role of the brain's plasticity during recovery. Additionally, LGI shows promise as a prognostic marker, with lower baseline values predicting poorer outcomes. Future research should prioritise integrating diverse cortical indices like LGI, AMC, SD, and SW in larger, longitudinal studies to enhance the robustness of findings and identify reliable biomarkers. [6] Defining standardized research protocols is also crucial for improving data comparability.

## Supplementary Information

Below is the link to the electronic supplementary material.

Supplementary file1 (DOCX 91 KB)

## Data Availability

All data are available within the article and supplementary materials. Additional information can be obtained from the corresponding author upon reasonable request.
