# Supplementary material for: Cortical complexity in eating disorders: a systematic review and qualitative synthesis
Source: Eur Arch Psychiatry Clin Neurosci. 2025 Apr 30;276(4):1941–57. doi: 10.1007/s00406-025-02001-3 (PMC13233882; doi:10.1007/s00406-025-02001-3)
Supplement: Supplementary file 1 — Supplementary file1 (DOCX 91 KB) [file 406_2025_2001_MOESM1_ESM.docx]

**Supplementary Material**

**Cortical complexity in Eating Disorders: a systematic review and qualitative meta-analysis**

**Running title: Cortical complexity in EDs**

Enrico Collantoni^1,2*^, Gianni Pessotto^1^, Valentina Meregalli^3^, Christopher R. Madan^4^, Alessandro Miola^1^, Giammarco Cascino^5^, Alessio Maria Monteleone^6^, Angela Favaro^1,2^

**Affiliations:**

1 Department of Neurosciences, University of Padua, Padova, Italy

2 Padua Neuroscience Center, University of Padua, Padova, Italy

3 Department of General Psychology, University of Padua, Padova, Italy

4 School of Psychology, University of Nottingham, Nottingham, UK.

5 Department of Medicine, Surgery and Dentistry, "Scuola Medica Salernitana", University of Salerno, Salerno, Italy

6 Department of Psychiatry, University of Campania L. Vanvitelli, Naples, Italy

***Corresponding author:** Enrico Collantoni – enrico.collantoni@unipd.it tel. +390498218175

Address: Department of Neurosciences, University of Padua, Via Giustiniani, 2 - 35128 Padova

Contents:

Supplementary Methods

**S.1. Supplementary Methods**

**S.1.1. Quality assessment**

All the studies were evaluated by two independent reviewers for quality (GP, EC) using the Imaging Methodology Quality Assessment Checklist (adapted fromStrakowski et al., 2000) based on the following parameters: subjects, imaging acquisition and analysis, and results and conclusions (Table S.1). All the studies demonstrated high quality; in fact, each study received a score of 8.5 or higher (see Figure S.1 for details)

**Table S.1. Imaging Methodology Quality Assessment Checklist.**

|  | **Category 1: Subjects** | **Score** (0/0.5/1) |
| --- | --- | --- |
| 1 | People with eating disorders were evaluated, specific diagnostic criteria were applied, and demographic data was reported |  |
| 2 | Healthy comparison subjects were evaluated, psychiatric and medical illnesses were excluded and demographic data was reported |  |
| 3 | Important variables (e.g. age, gender, BMI, total brain meseaure) were checked, either by stratification or statistically |  |
| 4 | Sample size per group > 7 |  |
|  | **Category 2: Methods for image acquisition and analysis** |  |
| 5 | All neuroanatomic measurements were taken without considering group assignment or subject identity |  |
| 6 | Magnet strength > 1T |  |
| 7 | The imaging technique used was clearly described so that it could be reproduced |  |
| 8 | Measurements were clearly described so that they could be reproduced |  |
|  | **Category 3: Results and conclusions** |  |
| 9 | Statistical parameters for significant, and important non-significant, differences were provided |  |
| 10 | Conclusions were consistent with the results obtained and the limitations were discussed |  |
|  | **TOTAL** | /10 |

The score for each item ranged from 0 to 1, where 0.5 was assigned when criteria were partially met. This qualitative rating was aimed at describing the methodological limitation of published studies to help the reader in weighting the importance of the findings of a study.

| **Author** | **Item 1** | **Item 2** | **Item 3** | **Item 4** | **Item 5** | **Item 6** | **Item 7** | **Item 8** | **Item 9** | **Item 10** | **Total score** |
| --- | --- | --- | --- | --- | --- | --- | --- | --- | --- | --- | --- |
| Favaro et al., 2015 | 1 | 0,5 | 1 | 1 | 1 | 1 | 1 | 1 | 1 | 1 | 9.5 |
| Schultz et al., 2017 | 1 | 0.5 | 1 | 1 | 1 | 1 | 1 | 1 | 1 | 1 | 9.5 |
| Bernardoni et al., 2018 | 1 | 1 | 1 | 1 | 1 | 1 | 1 | 1 | 1 | 1 | 10 |
| Miles et al., 2018 | 1 | 0.5 | 1 | 1 | 1 | 1 | 1 | 1 | 1 | 1 | 9.5 |
| Collantoni et al., 2019 | 1 | 1 | 1 | 1 | 1 | 1 | 1 | 1 | 1 | 1 | 10 |
| Leppanen et al., 2019 | 1 | 0.5 | 1 | 1 | 1 | 1 | 1 | 1 | 1 | 1 | 9.5 |
| Nickel et al., 2019 | 1 | 1 | 1 | 1 | 1 | 1 | 1 | 1 | 1 | 1 | 10 |
| Cascino et al., 2020 | 1 | 0.5 | 1 | 1 | 1 | 1 | 1 | 1 | 1 | 1 | 9.5 |
| Collantoni et al., 2020 | 1 | 1 | 1 | 1 | 1 | 1 | 1 | 1 | 1 | 1 | 10 |
| Collantoni et al., 2021 | 1 | 0.5 | 1 | 1 | 1 | 1 | 1 | 1 | 1 | 1 | 9.5 |
| Halls et al., 2022 | 0.5 | 0.5 | 1 | 1 | 1 | 1 | 1 | 0.5 | 1 | 1 | 8.5 |
| Li et al., 2023 | 1 | 1 | 1 | 1 | 1 | 1 | 1 | 1 | 1 | 1 | 10 |
| Collantoni et al., 2024 | 1 | 0.5 | 1 | 1 | 1 | 1 | 1 | 1 | 1 | 1 | 9.5 |

**Figure S.1. Quality assessment of ED studies on cortical complexity**

**References:**

Bernardoni, F., King, J. A., Geisler, D., Birkenstock, J., Tam, F. I., Weidner, K., Roessner, V., White, T., & Ehrlich, S. (2018). Nutritional Status Affects Cortical Folding: Lessons Learned From Anorexia Nervosa. *Biological Psychiatry*, *84*(9), 692–701. https://doi.org/10.1016/J.BIOPSYCH.2018.05.008

Cascino, G., Canna, A., Monteleone, A. M., Russo, A. G., Prinster, A., Aiello, M., Esposito, F., Salle, F. Di, & Monteleone, P. (2020). Cortical thickness, local gyrification index and fractal dimensionality in people with acute and recovered Anorexia Nervosa and in people with Bulimia Nervosa. *Psychiatry Research. Neuroimaging*, *299*. https://doi.org/10.1016/J.PSCYCHRESNS.2020.111069

Collantoni, E., Alberti, F., Dahmen, B., von Polier, G., Konrad, K., Herpertz-Dahlmann, B., Favaro, A., & Seitz, J. (2024). Intra-individual cortical networks in Anorexia Nervosa: Evidence from a longitudinal dataset. *European Eating Disorders Review : The Journal of the Eating Disorders Association*, *32*(2), 298–309. https://doi.org/10.1002/ERV.3043

Collantoni, E., Madan, C. R., Meneguzzo, P., Chiappini, I., Tenconi, E., Manara, R., & Favaro, A. (2020). Cortical Complexity in Anorexia Nervosa: A Fractal Dimension Analysis. *Journal of Clinical Medicine*, *9*(3). https://doi.org/10.3390/JCM9030833

Collantoni, E., Madan, C. R., Meregalli, V., Meneguzzo, P., Marzola, E., Panero, M., D’Agata, F., Abbate-Daga, G., Tenconi, E., Manara, R., & Favaro, A. (2021). Sulcal characteristics patterns and gyrification gradient at different stages of Anorexia Nervosa: A structural MRI evaluation. *Psychiatry Research. Neuroimaging*, *316*. https://doi.org/10.1016/J.PSCYCHRESNS.2021.111350

Collantoni, E., Meneguzzo, P., Tenconi, E., Manara, R., & Favaro, A. (2019). Small-world properties of brain morphological characteristics in Anorexia Nervosa. *PloS One*, *14*(5). https://doi.org/10.1371/JOURNAL.PONE.0216154

Favaro, A., Tenconi, E., Degortes, D., Manara, R., & Santonastaso, P. (2015). Gyrification brain abnormalities as predictors of outcome in anorexia nervosa. *Human Brain Mapping*, *36*(12), 5113–5122. https://doi.org/10.1002/HBM.22998

Halls, D., Leppanen, J., Kerr-Gaffney, J., Simic, M., Nicholls, D., Mandy, W., Williams, S., & Tchanturia, K. (2022). Examining the relationship between autistic spectrum disorder characteristics and structural brain differences seen in anorexia nervosa. *European Eating Disorders Review : The Journal of the Eating Disorders Association*, *30*(5), 459–473. https://doi.org/10.1002/ERV.2910

Leppanen, J., Sedgewick, F., Cardi, V., Treasure, J., & Tchanturia, K. (2019). Cortical morphometry in anorexia nervosa: An out-of-sample replication study. *European Eating Disorders Review : The Journal of the Eating Disorders Association*, *27*(5), 507–520. https://doi.org/10.1002/ERV.2686

Li, W., Wang, M., Wu, G., Wang, J., Li, X., Yang, Z., Chen, Q., Yang, Z., Li, Z., Zhang, P., Tang, L., & Wang, Z. (2023). Exploration of the relationships between clinical traits and functional connectivity based on surface morphology abnormalities in bulimia nervosa. *Brain and Behavior*, *13*(4). https://doi.org/10.1002/BRB3.2930

Miles, A. E., Voineskos, A. N., French, L., & Kaplan, A. S. (2018). Subcortical volume and cortical surface architecture in women with acute and remitted anorexia nervosa: An exploratory neuroimaging study. *Journal of Psychiatric Research*, *102*, 179–185. https://doi.org/10.1016/J.JPSYCHIRES.2018.04.010

Nickel, K., Joos, A., Tebartz van Elst, L., Holovics, L., Endres, D., Zeeck, A., & Maier, S. (2019). Altered cortical folding and reduced sulcal depth in adults with anorexia nervosa. *European Eating Disorders Review : The Journal of the Eating Disorders Association*, *27*(6), 655–670. https://doi.org/10.1002/ERV.2685

Schultz, C. C., Wagner, G., de la Cruz, F., Berger, S., Reichenbach, J. R., Sauer, H., & Bär, K. J. (2017). Evidence for alterations of cortical folding in anorexia nervosa. *European Archives of Psychiatry and Clinical Neuroscience*, *267*(1), 41–49. https://doi.org/10.1007/S00406-015-0666-1

Strakowski, S. M., Delbello, M. P., Adler, C., Cecil, K. M., & Sax, K. W. (2000). Neuroimaging in bipolar disorder. *Bipolar Disorders*, *2*(3), 148–164. https://doi.org/10.1034/J.1399-5618.2000.020302.X
